# Rethinking engagement

**DOI:** 10.1192/bjb.2018.55

**Published:** 2019-02

**Authors:** David Gilbert

**Affiliations:** *In*Health Associates, UK; Sussex Musculoskeletal Partnership (Central), UK

## Abstract

People affected by health conditions bring insights and wisdom to transform healthcare – ‘jewels from the caves of suffering'. Yet traditional patient and public engagement relies on (child–parent) feedback or (adolescent–parent) ‘representative' approaches that fail to value this expertise and buffers patients' influence. This editorial outlines the emergence of ‘patient leadership' and work in the Sussex Musculoskeletal Partnership, its patient director (the first such role in the National Health Service) and a group of patient/carer partners, who are becoming equal partners in decision-making helping to reframe problems, generate insight, shift dynamics and change practice within improvement and governance work.

**Declaration of interest:**

David Gilbert is Director of *In*Health Associates Ltd, a small consultancy organisation that supports patient and public engagement.

‘*Some important principles are becoming well established: these are the antiseptic power of transparency, a commitment to both personal and shared responsibility and a renewed engagement with patients and the public.*’ Rethinking Regulation. (p. 21)^1^

Calls for patient engagement in mental healthcare seem ubiquitous. There appears to be a consensus that people who use services must now work alongside staff to identify solutions to current healthcare challenges. However, if we are serious about this renewed engagement, we need to think carefully. The task is fourfold. First, we need to learn to value what patients can bring, which I call ‘seeing patients as partners’. Second, we need to change how engagement is done, by rethinking the engagement process. Third, we need to support people's capabilities to better work together. This includes developing the right skills. Finally, we must develop new opportunities for patients to influence decision-making by creating new roles.

## Valuing what patients can bring – patients as partners

People who have been affected by life-changing illness, injury or disability can help. We bring jewels of wisdom and insight from the caves of suffering^2^ – we know intimately what it is like to feel vulnerable and powerless, the effect of pain and suffering on lives, the primacy of healing relationships in care and what good and poor services look like. This combination of vision, humanity and integrity are essential components of high-quality leadership.

About 20 years ago, I was sitting on a psychiatric ward with nothing to do – the lunch had been awful, the occupational therapist had been sacked (so no activities that afternoon) and the ward seemed full of screaming folk. A doctor strolled onto our bay and gave a perfunctory nod before gingerly pulling on the curtain rail beside my bed. Even in my disturbed state, I could see his behaviour was odder than mine. I asked him what he was doing. ‘Just checking to see if you could do anything stupid’, he replied, before walking back down the corridor. I was left contemplating the sudden and unintended addition to my range of ‘treatment’ options.

Fast forward 10 years: I was Head of Patients and the Public at the Commission for Health Improvement, the health inspectorate at the time (predecessor of the Healthcare Commission and Care Quality Commission). I was reading the National Patient Safety Agency standards on mental healthcare (though I don't recall which one, exactly). One of them was to decrease in-patient psychiatric suicides to zero by…. removing all non-collapsible curtain rails. I remembered that doctor who had checked my curtain rail. At about that time, three fellow in-patient friends of mine died. One had choked to death on her food while unsupervised, after she had left the psychiatric unit and gone to a nursing home (she had earlier been paralysed from the neck down through a failed suicide attempt). One had gone to his caravan and hanged himself. And one had drowned himself in the local reservoir.

All those deaths had occurred away from the in-patient environment, so the unit would have passed its inspection by having removed ligature points. It might also have been congratulated on its risk policies. This was ‘hitting the target and missing the point’. The unit had responded to the caravan and reservoir deaths by locking the doors at 20.00 h. This deprived me of my one visitor, a local chaplain who I could only get to see at 21.00 h. Nights became a pressure cooker of aggravated emotions – the consequence of this lack of trust and forced containment felt unsafe. I wonder whether dialogue between us in-patients and staff about what makes for a safe environment might have saved my friends.

Later, as part of a Collaboration for Leadership in Applied Health Research and Care Fellowship (North-West London), I undertook qualitative research that revealed seven benefits of high-quality patient engagement in improvement work (https://futurepatientblog.com/2015/03/22/seven-things-that-patients-bring-the-benefits-of-patients-as-partners-for-change/).^3^ I found several distinct benefits. Having patients as partners in the room will reframe the problems to be tackled. Focusing on what matters to patients leads to pathway redesign with better awareness of access issues, information and explanations needed at each stage, more humanity and better customer care. Patient engagement also promotes the finding of potential solutions to problems. Patients have the passion, insight, imagination and freedom from institutionally limited thinking to ask ‘What if…’? They also widen the array of options for improvement and change.

This process necessarily leads to changed relationships. With patients in the room, others are given permission to explore. Dynamics change, trusted relationships develop, people work together and move beyond us–them conversations to dialogue. Shared decision-making emerges. There are also individual benefits. Patients feel more confident, develop new skills and build on those skills buried during times of illness – and come to feel better. Staff gain too. Morale is lifted as conversations become about what can be done, they can feel that we are truly all in this together. This sort of work rehumanises healthcare. It should be noted that this approach may also help staff who suffer emotional distress. In mental health (and perhaps beyond) there has never been a better time for people with health problems to work with staff (clinicians and support staff alike) to change and improve approaches to well-being and to explore our common humanity, rethink professional role boundaries (the ‘them and us’ mentality) and approaches to safety and risk.

The result is better quality decisions. If people know why decisions have been made and been part of that process, this generates trust, confidence and it becomes easier to build consensus. This has deep implications for transparency, governance and accountability. I have seen and heard about dozens of changes in policy and practice as a result of patients being partners in improvement work: making guidelines more flexible, better ways to tackle access and equalities, tackling attitudes and behaviours, different ways of meeting unmet need, the list is endless. There are even benefits beyond the project. When people see the advantages of patients as partners for improvement and change in one area, they will help spread it to others. It is a virtuous cycle with implications for scaling up improvement processes, spreading good practice and sustainability.

## Changing how engagement is done

The traditional approaches to involving or engaging patients do not work, and so we fail to value the jewels offered or to change the ‘currency’ of healthcare toward what matters. Patient and public engagement, as traditionally conceived, buffers power by distancing patients from decision-making. Thus, it maintains the status quo by preserving the institutional authority of professional system leaders. Ironically, when engagement is seen to fail, as it often does, this can be attributed to the lack of value that patients bring rather than to faulty mechanisms. The engagement industry focuses largely on inputs, activities and processes (the methods of gathering data, how to capture views, etc.) over impact and outcomes.

The approaches and methods used rely on two main styles. The first is that of feedback: patients are invited to fill in questionnaires, attend focus groups or tell their stories (if they are lucky) at board meetings or the like. The focus is what happened to them in the past, mostly about their experience of services (rather than living with a condition, or about their lives beyond the institutional scope of interest), and the meaning of their data is left to professionals to assess through their own lenses based on their own assumptions and often narrow institutionalised thinking (often what is seen as feasible rather than necessary). Patients are not permitted to eyeball the data or bring their own interpretations to it, let alone be partners in decisions about what to do. This feedback approach mirrors traditional medical paternalistic models – you tell us the symptoms and we will provide the diagnosis and treatment. It is stuck in child–parent mode.

The second style is scrutiny. Whenever there is a governance committee, an advisory group or the like, the call goes out for a lay representative. I know a patient and public involvement lead who likened her role to that of ‘lay rep pimp’. Without clarity of role, support or training, a representative is expected to bring the patient perspective to the decision-making table. I was once asked ‘so David, what do patients think?’. What, all of them? I thought. In search of credibility and leaning on what we know, we tell our stories, and half the people in the room applaud this ‘telling truth to power’ and the other half fall asleep (‘another patient with an axe to grind’ or ‘personal agenda’ they mutter later in the corridors). If we wise up and come to the table next time wearing a suit and tie, brandishing data, those that were awake last time fall asleep and accuse us of ‘going native’. I have written about this representative trap in more detail elsewhere.^4^

The consequence of failed representational mechanisms is that committees lapse into a default ‘us and them’ mode. Frustrated, marginalised and unprepared representatives start finger-wagging or fall silent. This is adolescent–parent style engagement. If we are serious about partnership, then we need to overhaul the engagement industry.

## Supporting people's capabilities – the emergence of Patient Leaders

The past few years have seen the rise of new forms of engagement such as online dialogue, experience-based co-design, health champions, peer support and the like. Mark Doughty and I founded the Centre for Patient Leadership to support patients (those with life-changing illness injury or disability, and/or with long-term conditions) to be influential change agents. CPL trained over 1000 patients to develop the capabilities to work with professionals as equal partners. Further information on patient leadership is available online (http://www.inhealthassociates.co.uk/patient-leadership-articles-and-reports/).

Patient Leaders are those who have been affected by life-changing illness, injury or disability and want to work with others in partnership to influence change. This can, of course, include carers. They can have many roles. Some are entrepreneurs like Michael Seres, a patient who had undergone a bowel transplant and then invented a Bluetooth sensor-enabled colostomy bag that does not overflow. He has also led the way on remote-access technology to allow people to communicate with their clinicians, and is Chief Executive of his own company, Health 11. Others are campaigners or activists, online dialogue specialists, improvement advisors or help organisations as governors or are part of inspection processes. They work at local, regional and national levels. (I am writing a book entitled ‘*The Jewel Merchants*’, which will be published in 2019, that is based on the stories of 15 such people, including Alison Cameron, Ceinwen Giles, Dominic Makuvachuma-Walker, Patrick Ojeer and Sibylle Erdmann).

There needs to be wider investment in skills development; indeed, one might question why tens of millions of pounds is spent investing in the capabilities of managerial and clinical leadership, and none on this emerging army of people who could – and I think will – regenerate healthcare. There is still a widespread assumption that system leaders are professionals, but for Patient Leaders to achieve their full potential, they also need the learning and development that enables them to be true leaders.

Finally, there has to be an equal emphasis on creating the right opportunities, for example, in governance, research and audit, service improvement and training and education. This could be at a local or national level, but needs to be where professionals are willing and able to work as partners too. Opportunities must also be created at a senior level. In much the same way that it is not considered appropriate that a service purporting to deliver ‘women-centred care’ is led entirely by men, in a few years' time it will seem odd that we have ever had a patient-centred National Health Service (NHS) run entirely by clinical and managerial leaders.

The Sussex Musculoskeletal (MSK) Partnership (Central Sussex) receives referrals from general practitioners of people who have joint, muscle or bone problems. The service stretches from Brighton and Hove, through mid-Sussex and Horsham to Crawley. Clinicians screen referrals, and many are offered an appointment at our specialist clinics, with advanced MSK practitioners or physiotherapists (often working alongside consultants and others, such as psychologists).

The Partnership is a lead accountable provider. It comprises the Sussex NHS Community Trust, Sussex Partnership Trust, HERE (a social enterprise) and The Horder Centre (a charity). In Autumn of 2015, three clinical commissioning groups pooled a total of £50 m per year for 5 years to us, so we could run a better system for people who use services. We want to get it right first time, so that people do not have to go here, there and everywhere for different diagnostic and treatment interventions. And we, like the NHS rhetoric always says, want patients to be at the heart of what we do.

## New opportunities – the patient director and patient partners

The Sussex MSK Partnership (Central) made a brave decision to appoint the first patient director – someone who has had experience of a life-changing illness, injury or disability (in my case, mental health problems) and can harness these experiences at senior decision-making levels. This role ensures that patient leadership is embedded at a senior level, within an executive team that includes a clinical director and managing director. This models shared decision-making at corporate level. The patient director's role is to help the Partnership focus on what matters. This includes embedding patient-centred cultures, systems and processes such that they become ‘hardwired’ and making sure we learn from, and act on what patients’ think about services. The patient director will also support patients to enable them to be influential and valued partners in decision-making.

Being a patient director has enabled me to experiment with a different approach to engagement. For example, we have eight patient and carer partners. They bring professional and personal wisdom alongside their experiences of using our services. Patient and carer partners are not representatives or there to provide feedback, but are ‘critical friends’ who check assumptions, ask questions, provide insights into reframing issues or identifying problems, change dynamics and model collaborative leadership.

My role is to broker opportunities in improvement or governance and support them to ensure they have the capacity and capability to be effective. Patient and carer partners augment other involvement and feedback work. This work has been developed during a period of intense operational pressures. During the past 3 years, the Partnership has transformed the way MSK services are delivered (through a lead accountable provider model – see box) and patient partners have been alongside as we have done so. We wanted them to be partners in every MDT that oversees quality in each of our musculoskeletal pathways (orthopaedics, rheumatology, pain management and physiotherapy).

The first step was for the patient director to identify opportunities for meaningful engagement and ensure their presence in improvement and redesign work. Then, to be clear that they were more than storytellers or to feed back on their experiences (we had other data for that), they stayed in the room, proving themselves well able to reframe problems, generate new solutions, model collaborative leadership and shift dynamics. Patient partners have been involved in seven major improvement programmes: pain services redesign, fibromyalgia pathways, development of patient reported outcome measures, plans for shared decision-making, administrative systems, support for receptionists and call handlers and integration of physical and mental health provisions.

An early experience helped us to demonstrate benefits. We were discussing how to communicate with patients about booking appointments. We were receiving lots of calls to cancel or change inconvenient appointments that we had booked for people. A woman who had been through our service, told us that our team phoned at inconvenient times to book appointments. She suggested that, instead, we send opt-in appointment letters and put her in the driving seat. Let her phone back when she had her diary in front of her and she could plan out her week. We experimented with the idea and it was successful, with patients and call handlers alike delighted with how it worked. If this approach were rolled out, we would save an estimated 3500 cancelled appointments per year.

Slowly, they have become trusted equals. It has not been easy and is dependent on clarity of role, shared understanding of purpose, demonstrating benefits and the perennial time, money, space, trust… all things the NHS has precious little of. We are ready for the next step – for partners to move from an improvement role into a more steady-state governance role. However, given inevitable resource constraints, we realise that we cannot support two patient partners in each of our eight MDTs. So patient partners have come up with a different approach: the idea of a pilot special MDT. This might model the sort of reflective dialogue they want to demonstrate and focus on issues of quality and patient experience. We would evaluate the work and see whether it could be a model for other pathways. Members of the hip and knee pathway MDT seem keen on the idea, and next month we will be talking to them about how it could work.

We will also discuss whether and how we can ensure that patients are a part of regular MDTs. Several other clinical leads are watching this experiment with interest, and it could pave the way for a different model of reflective governance across the Partnership. It has taken 3 years for this work to take off – the role of patient director is still novel, and this particular model of patient partnership is an experiment. It has taken months of building relationships, doing the ground work and making the case for a different model of engagement.

In the current frenzy surrounding NHS policy and practice, it is worthwhile remembering that long-term improvements take time, space and trust. There are no quick fixes. Our work in Sussex demonstrates one novel approach to the challenges of rethinking engagement. It is predicated on the four steps necessary to renew engagement – to value what people bring, establish different mechanisms for dialogue, to develop people's capabilities and provide new opportunities for the new breed of patient (or carer) leaders. Looking back, I now wonder what might have happened if a patient director had been around when I was on the psychiatric unit. Might my three friends still be alive?

## About the author

**David Gilbert** is Director of *In*Health Associates and Patient Director at Sussex Musculoskeletal Partnership (Central Sussex), UK.
